# Experience of a Neuro-Emergency Expert in the Emergency Department during One Year of the COVID-19 Pandemic

**DOI:** 10.3390/ijerph18189461

**Published:** 2021-09-08

**Authors:** Yong-Won Jung, Sang-Ook Ha, Jin-Hyouk Kim, Won-Seok Yang, Young-Sun Park

**Affiliations:** 1 Department of Emergency Medicine, Hallym University Medical Center, Hallym University Sacred Heart Hospital, 22, Gwanpyeong-ro 170 beon-gil, Dongan-gu, Anyang 14068, Korea; ddangee88@gmail.com (Y.-W.J.); stone1st@naver.com (W.-S.Y.); ndyspark@hanmail.net (Y.-S.P.); 2 Department of Neurology, Hallym University Medical Center, Hallym University Sacred Heart Hospital, 22, Gwanpyeong-ro 170 beon-gil, Dongan-gu, Anyang 14068, Korea

**Keywords:** emergency department, neurologist, stroke, vertigo

## Abstract

We aimed to evaluate the overall clinical characteristics of patients treated by a neuro-emergency expert dedicated to the emergency department (ED) as an attending neurologist during the COVID-19 pandemic. We included adult patients who visited the ED between 1 January and 31 December 2020 and were treated by a neuro-emergency expert. We retrospectively obtained and analyzed the data on patients’ clinical characteristics and outcome. The neuro-emergency expert treated 1155 patients (mean age, 62.9 years). The proportion of aged 18–40 years was the lowest, and the most common modes of arrival were public ambulance (50.6%) and walk-in (42.3%). CT and MRI examinations were performed in 94.4 and 33.1% of cases, respectively. The most frequent complaints were dizziness (31.8%), motor weakness (24.2%), and altered mental status (15.8%). The ED diagnoses were acute ischemic stroke (19.8%), benign paroxysmal positional vertigo (14.2%), vestibular neuritis (9.9%), and seizure (8.8%). The mean length of stay in the ED was 207 min. Of the patients, 55.0% were admitted to the hospital, and 41.8% were discharged for outpatient follow-up. Despite the longer stay and the complexity and difficulty of neurological diseases during the COVID-19 pandemic, the accurate diagnosis and treatment provided by a neuro-emergency expert can be presented as a good model in the ED.

## 1. Introduction

Neurological symptoms in emergency department (ED) settings vary on a spectrum of presentation and diagnosis. Approximately 9–15% of patients admitted to the ED have neurological symptoms, accounting for a significant proportion of the patients visiting the ED [1,2,3]. However, neurology is still considered a complex and unknown area for most doctors [4]. The aging population, growing demand for neurological management, new developments in neuroimaging tests, and advanced treatments for acute stroke have increased the number and complexity of neurological emergency cases [5]. Neurological diseases are the most common cause of disability and the second leading cause of death; consequently, the responsibility and burden of emergency physicians have increased [6].

As the ongoing coronavirus disease 2019 (COVID-19) pandemic has changed healthcare systems worldwide, especially in neurology, COVID-19 has been correlated with a reduction in admissions for transient, mild, and moderate strokes [7,8]. However, to our knowledge, there have been no reports on the overall characteristics of patients who visited the ED with neurological symptoms during the COVID-19 pandemic until recently; therefore, assessing and treating such patients is more challenging for emergency physicians.

Our study aimed to evaluate the overall clinical characteristics of patients treated by a neuro-emergency expert who resided with an emergency physician in the ED station for a year during the COVID-19 pandemic.

## 2. Materials and Methods

### 2.1. Study Design and Patient Selection

The study was conducted at the ED of an urban academic tertiary-care hospital in South Korea. This 900-bed facility has a regional emergency medical center, as designated by the government, and receives approximately 75,000 ED visits annually. Our hospital is the only university-affiliated and regional central hospital providing medical services to one million people in the surrounding four urbanized cities. In the ED, a neuro-emergency expert was responsible for the emergency care of patients with neurological symptoms. Through a systematic review of electronic medical records, our study included adult patients (≥18 years old) who visited the ED between 1 January and 31 December 2020.

### 2.2. Neuro-Emergency Expert’s Role and ED Process for Patients with Neurological Symptoms

Our ED has been operating a neuro-emergency medicine (NEM) project. The neuro-emergency expert had more than 18 years of practical experience in the clinical field and oversaw treatment in the ED from 08:00 to 18:00 on weekdays. A patient arriving at the ED with a complaint of neurological symptoms stayed with an on-duty emergency physician who began an intervention in the early stage. The physician’s primary role was to treat typical patients within the neurology department and was responsible for ED consultations for patients admitted to other departments, including internal medicine. At other times, an emergency physician and neurology resident were in charge of the treatment for patients with neurologic symptoms. Among the patients who visited our ED between 18:00 and 08:00, some patients had undetermined disposition or waited for hospitalization in the ED until the neuro-emergency expert went to work at 08:00 the next day. These patients were included in the present study and classified according to time of ED visit.

Patients admitted to the ED for neurological symptoms were evaluated for clinical severity using the Korean Triage and Acuity Scale (KTAS) in the triage room. Patients with a golden time limit for acute stroke or status epilepticus were directly treated by the neuro-emergency expert. In contrast, patients with non-emergent neurological symptoms were first treated by emergency physicians, who requested neurological consultation from the neuro-emergency expert, if deemed necessary.

### 2.3. Data Collection

We reviewed the enrolled patients’ EMRs to retrospectively obtain and analyze data on their baseline characteristics (age, sex, mode and route of arrival, and time of ED visit), diagnostic modality, KTAS level, chief complaint, ED diagnosis, and clinical outcome (length of stay (LOS) and ED disposition).

The KTAS, a classification tool for evaluating patient severity in the ED, was developed in 2012 by modifying the Canadian Triage and Acuity Scale to fit the medical situation in Korea [9]. This system classifies patients into the following five grades: level 1, resuscitation; level 2, emergency; level 3, urgent; level 4, less urgent; and level 5, non-urgent.

### 2.4. Statistical Analyses

Categorical variables were expressed as counts and percentages, and continuous variables as means and two standard deviations. All analyses were performed using SPSS version 18.0.0 for Windows (IBM, Armonk, NY, USA).

## 3. Results

### 3.1. Baseline Characteristics and KTAS Level of the Enrolled Patients

**Figure 1:**
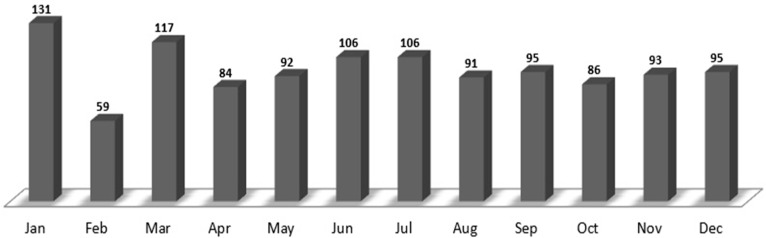
The monthly number of patients treated by a neuro-emergency expert in the emergency department between 1 January and 31 December 2020.

The baseline characteristics of the enrolled patients and the monthly trends are summarized in Table 1 and Figure 1. Among the 53,932 patients admitted to the ED between 1 January and 31 December 2020, 1155 patients (51.3% males and 48.7% females; mean age, 62.9 years) were treated (average, 96 patients/month) by the neuro-emergency expert. January and February had the highest (131) and lowest (59) number of patients, respectively. Additionally, the 60–80-year and 18–40-year age groups had the highest (480 (41.6%)) and lowest (108 (9.4%)) number of patients, respectively. Public ambulance (584 (50.4%)) was the most commonly used mode of arrival, followed by walking in (488 (42.3%)). A total of 997 (86.3%) patients visited the ED, whereas 158 (13.7%) patients were transferred from other hospitals.

**Figure 2:**
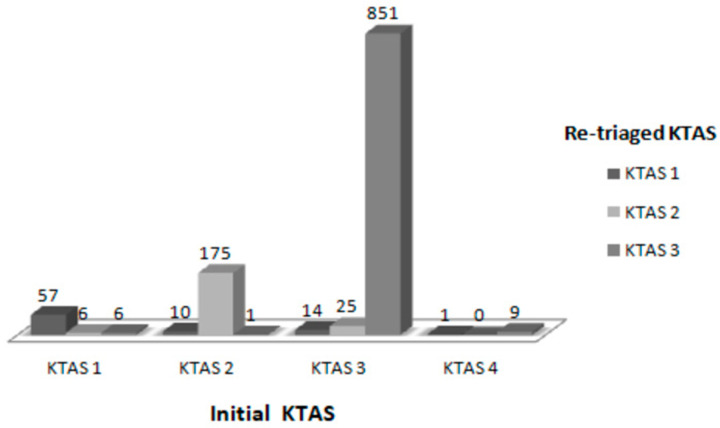
The initial Korean Triage and Acuity Scale (KTAS) and re-triaged KTAS findings.

Based on the initial KTAS severity classification, 69 patients were classified as level one, 186 patients as level two, 890 patients as level three, and 10 patients as level four. The subsequent re-triage identified that 50 patients were under-triaged, and 13 patients were over-triaged (Figure 2).

### 3.2. Neurological Diagnostic Evaluation

Of the enrolled patients, 1090 (94.4%) underwent computed tomography (CT), and 382 (33.1%) underwent magnetic resonance imaging (MRI). Brain diffusion-weighted imaging (DWI) was the most frequent modality among the MRI examinations. In addition, an electroencephalogram (EEG) was performed in 26 (2.3%) cases, and cerebrospinal fluid (CSF) tapping was performed in 10 (0.9%) cases (Table 2).

### 3.3. Chief Complaint and ED Diagnosis

**Figure 3:**
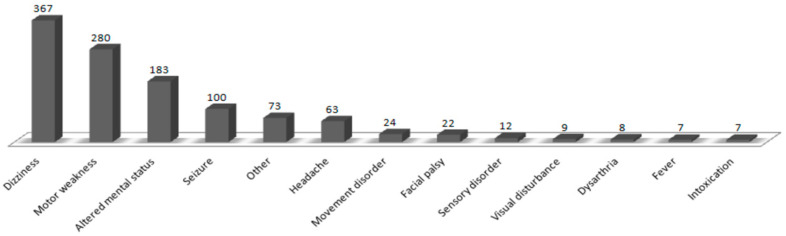
Chief complaints of the enrolled patients.

The most frequent complaints were dizziness, movement weakness, altered mental status, and seizures in 367 (31.8%), 280 (24.2%), 183 (15.8%), and 100 (8.7%) patients, respectively (Figure 3).

**Figure 4:**
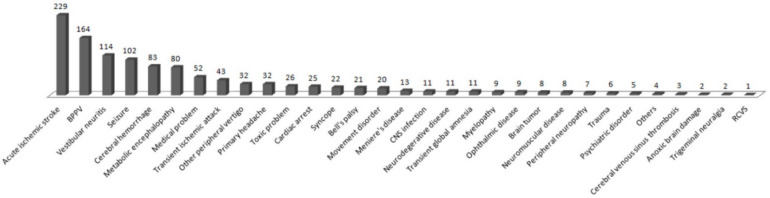
Emergency department diagnoses of the enrolled patients.

The diagnoses made by the neurologists in the ED included acute ischemic stroke, benign paroxysmal positional vertigo, vestibular neuritis, seizures, cerebral hemorrhage, and metabolic encephalopathy in 229 (19.8%), 164 (14.2%), 114 (9.9%), 102 (8.8%), 83 (7.2%), and 80 (6.9%) patients, respectively (Figure 4). Diagnoses grouped by age showed seizures and benign paroxysmal positional vertigo (BPPV) in 25 (23.1%) and 13 (12.0%) 13–40-year-old patients, respectively; BPPV and acute ischemic stroke (AIS) in 68 (19.0%) and 50 (14.0%) 40–60-year-old patients, respectively; AIS and BPPV in 109 (22.7%) and 75 (15.6%) 60–80-year-old patients, respectively; and AIS and metabolic encephalopathy in 66 (31.6%) and 23 (11.0%) patients older than 80 years, respectively (Table 3).

### 3.4. LOS and Disposition of the Enrolled Patients

The mean LOS in the ED was 207 min. According to the LOS, 159 (13.8%), 553 (47.9%), 225 (19.5%), and 218 (18.9%) patients stayed in the ER for <120, 120–240, 240–360, and >360 min, respectively (Table 4).

In terms of disposition, 635 patients (55%) were admitted to the hospital, of which 366 (31.7%) and 154 (13.3%) patients were admitted to the Department of Neurology and Internal Medicine, respectively. After discharge, 483 patients (41.8%) were determined for outpatient follow-up, and 13 patients (1.1%) were transferred to another hospital.

## 4. Discussion

To our knowledge, this is the first study based entirely on data from patients with neurological symptoms managed by a neurology expert dedicated to the ED. We presented the overall clinical characteristics of patients with neurological symptoms who visited the ED during the COVID-19 pandemic. The most common neurological symptom was dizziness, followed by motor weakness and altered mental status. In terms of the ED diagnosis, AIS was the most common neurological disease, followed by BPPV, vestibular neuritis, and seizures. In particular, seizure and BPPV were common diseases in patients under 60 years of age, and AIS, BPPV, and metabolic encephalopathy were common diseases in patients over 60 years of age.

### 4.1. Demographic and Baseline Characteristics

The mean age was 62.9 years, and the proportion of patients aged 18–40 years was the lowest. Only one such study has been conducted during the COVID-19 pandemic [10]; consequently, we compared our findings with studies conducted before the pandemic. Previous studies have shown that the number of patients visiting the ED for neurological symptoms is small among young people, and this trend continued during the COVID-19 pandemic. A public ambulance was the most common mode of arrival in our study, followed by a walk-in. During the study period, 27% of all the patients who visited our ED used a public ambulance. The proportion was significantly higher among patients with neurological symptoms in our study (50.6%) as well as in a study by Lindane et al. (40.9%) [3]. Our study’s higher ambulance utilization rate is understandable because dizziness, motor weakness, altered mental status, and seizures accounted for 80% of the chief complaints. However, further research is needed to determine whether the ambulance utilization rate increased during the COVID-19 pandemic. The number of patients transferred from other hospitals was small compared to direct visits. However, a direct comparison was limited by the lack of pre-pandemic data. Nevertheless, the difficulty of transferring between hospitals during the COVID-19 pandemic is frequently encountered in clinical fields. Since patients with an altered mental status cannot confirm the epidemiological and clinical features of COVID-19, the transfer proceeds after identifying negative test results for COVID-19 at the referral hospital.

### 4.2. Modalities for Neurological Evaluation

Of the patients enrolled in our study, 1090 (94.4%) underwent CT, which was remarkably higher than 37.6 and 65.5% in other studies [10,11], possibly due to differences in each country’s health care insurance policy. CT is the most frequently performed basic modality for the quick and accurate identification of acute hemorrhage, mass lesions, and a recent cerebral infarction with acute neurological symptoms. In our study, 382 (33.1%) patients underwent MRI examinations, which was relatively less than 66.9% of MRI examinations in the Covan et al. study, primarily because unnecessary MRI examinations were reduced in our ED by the neuro-emergency expert. Most of the MRI scans performed in the ED to differentiate acute ischemic stroke were brain DWI. In terms of acute stroke, lesion localization is often possible with clinical features and brain CT scans. Unlike CT, MRI has many limitations, such as time consumption, unstable airway and breathing, and the inability to cooperate. Especially, in the COVID-19 pandemic, there may be guidelines that an MRI exam, which take a long time, should be performed after confirming a negative COVID-19 test, depending on the medical institution, such as our emergency center. In these cases, there may be an advantage in that unnecessary MRI scans can be reduced and the length of stay in the ED has also been shortened according to the clinical judgement of the neuro-emergency expert.

In our study, EEG and CSF tapping were performed in 26 (2.3%) and 10 patients (0.9%), respectively. Our ED has been using a portable EEG since 2019, and although the test rate has decreased for infection control during the COVID-19 pandemic, the EEG test performed during the study period was 2.3%, which was approximately 25% of patients with seizures. Few studies have performed a portable EEG in the ED; Covan et al. and Hoyer et al. reported that EEG examinations were performed in 1 and 0.2% of patients, respectively. An EEG is particularly useful for treating patients with seizures, especially when evaluating treatment responses in real-time for patients with status epilepticus and helps choose or adjust the antiepileptic drug.

### 4.3. Chief Complaint and ED Diagnosis

The most frequent complaints were dizziness, movement weakness, altered mental status, and seizures. Although the order of the most common symptoms differs from previous studies [1,10,12], the first and second most common symptoms (dizziness and motor weakness) were the same. The diagnoses made most frequently by neuro-emergency experts in the ED were acute ischemic stroke, benign paroxysmal positional vertigo, vestibular neuritis, seizures, cerebral hemorrhage, and metabolic encephalopathy. The order of diagnosis frequency in other studies was AIS, seizure, and headache [1,5,10,13,14]; our results had similar AIS frequency but fewer cases of headache. Most cases of primary headache with no abnormal findings on brain CT scans were discharged by the ED physicians after controlling their symptoms and referred for outpatient treatment. Consequently, there were relatively fewer consultations with neuro-emergency experts. A relatively large number of patients reported dizziness compared with other studies. Dizziness cannot be diagnosed with a brain CT; therefore, it is difficult for emergency physicians to diagnose its cause accurately, and often professional treatment is necessary for central vertigo, requiring a neuro-emergency expert’s intervention.

A neuro-emergency expert in the ED enabled us to confirm various rare and serious ED diagnoses by making accurate and detailed diagnoses while directly treating the patients. In addition, we demonstrate differences in diagnosis by age and a wide spectrum of chief complaints (Table 3; Supplementary Materials Table S1), thus explaining why it is challenging to diagnose patients with neurological symptoms.

### 4.4. Clinical Outcomes of Patients during the COVID-19 Pandemic

Early intervention by a neurologist is needed to diagnose patients with neurologic symptoms at an early stage and provide accurate initial treatment in the ED. Studies have demonstrated that a dedicated neurologist in the ED reduced the length of stay and the hospital admission rate [3,11].

The mean LOS in our ED (207 min) was longer than the LOS reported by Linden et al. (156 min) but shorter than the LOS reported by Hoyer et al. (250 min) and Hansen et al. (408 min) [1,3,12]. The most frequently observed LOS in our study was 120–240 min (553 patients, 47.9%), and 218 patients (18.9%) stayed in the ED for >360 min. Regardless of fever or respiratory symptoms, a negative COVID-19 test was confirmed in ED patients who required hospital admission before admitting them.

In terms of disposition, 55% of enrolled patients were admitted to the hospital, 41.8% were determined for outpatient follow-up, and 1.1% were transferred to another hospital. The admission rate was similar to that reported by Hoyer et al. and Linden et al. (50.0–57.7%) [1,3,10].

### 4.5. Strengths and Limitations

Our study has significant clinical relevance, as it reports the experience of having a neuro-emergency expert along with an emergency physician to treat patients in the ED. A system such as that in our ED to provide real-time, face-to-face treatment to patients with neurological symptoms in the ED is extremely rare, and only a few studies have investigated its utility [1,2,15]. Although the operating system varies by countries and hospitals, in most EDs, neurological diseases are treated by emergency physicians or neurology residents. Only several studies, the patients with neurologic symptoms in the ED were treated by a dedicated neurologist. In particular, Linden et al. operated a dedicated neurologist for a longer time (additionally during weekdays between 5 p.m. and 11 p.m., and during the weekends between 2 p.m. and 6 p.m.) than we did. Although there are limitations to generalizing these results to other EDs, there is also the aspect of helping other clinicians by sharing the experiences of a neuro-emergency physician

Our study has limitations. First, its single-center, retrospective study design may have introduced selection bias. Among the patients who visited the ED during weekdays, only those who received a neuro-emergency expert consultation were included in our study. However, since it was based on the experience of a single neuro-emergency expert and the data were collected for a period of one year, our results sufficiently reflescted the clinical situation. Furthermore, our study has limited generalizability as there are limited data on the COVID-19 pandemic causing changes in medical healthcare. Second, the sample size was not calculated, and only the patients who visited the ED over a period of one year were included. Third, the diagnosis made by a neuro-emergency expert in the ED may differ from the final diagnosis made at the time of hospital admission or in the outpatient clinic. Although not discussed, the final diagnosis changed in ten patients who were discharged from the ED (data not shown). Fourth, Moeller et al. reported that the concordance and discordance in the final diagnosis of ED physicians were 60.4 and 39.6%, respectively, and the discordance rate between ED physicians and neurologists was 35.7% [14]. Unfortunately, the data on the concordance rate of the final diagnosis of emergency physicians and neuro-emergency experts were not present in our study. Fifth, there was no comparison between before and after the neuro-emergency expert treatment engagement in the ED; therefore, the direct effect of the clinical outcome could not be confirmed. To overcome these limitations and generalizations, it is necessary to operate a system that can cover all neurologic diseases for 24 h a day, 7 days a week, and conduct a study setting a longer period, including the COVID-19 pandemic. In addition, more detailed and well-planned research is required to confirm the clinical advantages of the neuro-emergency expert compared to the previous general system.

## 5. Conclusions

The most common neurological symptom in the ED during the COVID-19 pandemic was dizziness, followed by motor weakness and altered mental status. In terms of ED diagnosis, AIS was the most common neurological disease, followed by BPPV, vestibular neuritis, and seizures. In particular, seizure and BPPV were common diseases in patients under 60 years of age, and AIS, BPPV, and metabolic encephalopathy were common diseases in patients over 60 years of age.

Given the longer LOS in the ED and the complexity and difficulty of treating neurological diseases during the COVID-19 pandemic, the accurate diagnosis and treatment provided by a neuro-emergency expert can be invaluable in the ED.

## Figures and Tables

**Table 1:** Baseline characteristics of the enrolled patients.

Variable	Enrolled Patients (*N* = 1155)
Age (years)	62.9 ± 16.5
18 ≤ age < 40	108 (9.4%)
40 ≤ age < 60	358 (31.0%)
60 ≤ age < 80	480 (41.6%)
80 ≤ age	209 (18.1%)
Sex	
Male	593 (51.3%)
Female	562 (48.7%)
Mode of arrival	
Walk-in	488 (42.3%)
Public ambulance	584 (50.6%)
Private ambulance	83 (7.2%)
Route of arrival	
Direct	997 (86.3%)
Transfer from other hospital	158 (13.7%)
Time of emergency department visit	
Day (08:00–16:00)	903 (78.2%)
Evening (16:00–24:00)	162 (14.0%)
Night (00:00–08:00)	90 (7.8%)

Each result is presented as either count (percentage) or mean ± two standard deviations.

**Table 2:** The type of neurological diagnostic evaluation performed in the enrolled patients.

Variable	Enrolled Patients (*N* = 1155)
CT	1090 (94.4%)
Brain non-contrast CT	1052 (91.1%)
Brain CT angiography	34 (2.9%)
Spine CT	4 (0.3%)
MRI	382 (33.1%)
Brain DWI only	329 (28.5%)
Brain MRI + MRA	3 (0.3%)
Brain MRA + DWI	5 (0.4%)
Brain MRI + DWI	1 (0.1%)
Brain MRI + MRA + DWI	21 (1.8%)
Brain MRI with CE	2 (0.2%)
Brain MRI with CE + MRA + DWI	11 (1.0%)
Brain MRI with CE + MRA + DWI + Perfusion	4 (0.3%)
Brain MRI with CE + Aortic arch angiography + DWI	1 (0.1%)
Brain MR venography	1 (0.1%)
Temporal/IAC with CE	1 (0.1%)
Spine MRI	3 (0.3%)
Electroencephalogram	26 (2.3%)
Cerebrospinal fluid tapping	10 (0.9%)
No work-up	42 (3.6%)

Results are presented as counts and percentages. CE, contrast enhancement; CT, computed tomography; DWI, diffusion-weighted imaging; ED, emergency department; IAC, internal auditory canal; MRA, magnetic resonance angiography; MRI, magnetic resonance imaging.

**Table 3:** Emergency department diagnoses according to the age group.

Rank	Age Group (in Years)			
	18 ≤ Age < 40 (*N* = 108)	40 ≤ Age < 60 (*N* = 358)	60 ≤ Age < 80 (*N* = 480)	80 ≤ Age (*N* = 209)
1	Seizure (25, 23.1%)	BPPV (68, 19.0%)	AIS (109, 22.7%)	AIS (66, 31.6%)
2	BPPV (13, 12.0%)	AIS (50, 14.0%)	BPPV(75, 15.6%)	Metabolic encephalopathy (23, 11.0%)
3	Primary headache (10, 9.3%)	Seizure (48, 13.4%)	Vestibular neuritis (53, 11.0%)	Seizure (15, 7.2%)
4	CNS infection (8, 7.4%)	Vestibular neuritis (47, 13.1%)	Cerebral hemorrhage (40, 8.3%)	Cerebral hemorrhage (14, 6.7%)
5	Medical problem (5, 4.6%)	Cerebral hemorrhage (25, 7.0%)	Metabolic encephalopathy (34, 7.1%)	Medical problem (14, 6.7%)

Values in parentheses after each diagnosis refer to the corresponding count and percentage of the total count in each age group. AIS, acute ischemic stroke; BPPV, benign paroxysmal peripheral vertigo; CNS, central nervous system.

**Table 4:** The length of stay and disposition of the enrolled patients.

Variables	Enrolled Patients (*N* = 1155)
LOS (in minutes)	207.0 (144.0–309.0)
LOS < 120	159 (13.8%)
120 ≤ LOS < 240	553 (47.9%)
240 ≤ LOS < 360	225 (19.5%)
360 ≤ LOS	218 (18.9%)
ED disposition	
Admission to a hospital department	635 (55.0%)
Neurology	366 (31.7%)
Internal Medicine	154 (13.3%)
Cardiothoracic Surgery	2 (0.2%)
Otolaryngology	4 (0.3%)
General Surgery	2 (0.2%)
Psychiatry	1 (0.1%)
Neurosurgery	94 (8.1%)
Orthopedic Surgery	6 (0.5%)
Pediatrics	1 (0.1%)
Spine Center	4 (0.3%)
Urology	1 (0.1%)
Outpatient Department for follow-up	483 (41.8%)
Transfer to another hospital	13 (1.1%)
DAMA discharge	21 (1.8%)
Death	3 (0.3%)

Each result is presented as either count (percentage) or median (25–75th percentiles). DAMA, discharge against medical advice; ED, emergency department; ICU, intensive care unit; LOS, length of stay.

## Supplementary Materials

The following are available online at https://www.mdpi.com/article/10.3390/ijerph18189461/s1, Table S1: Various spectrums of ED diagnosis according to the chief complaints.
