# Supplementary figures and images for: Experience of a Neuro-Emergency Expert in the Emergency Department during One Year of the COVID-19 Pandemic
Source: Int J Environ Res Public Health. 2021 Sep 8;18(18):9461. doi: 10.3390/ijerph18189461 (PMC8493796; doi:10.3390/ijerph18189461)

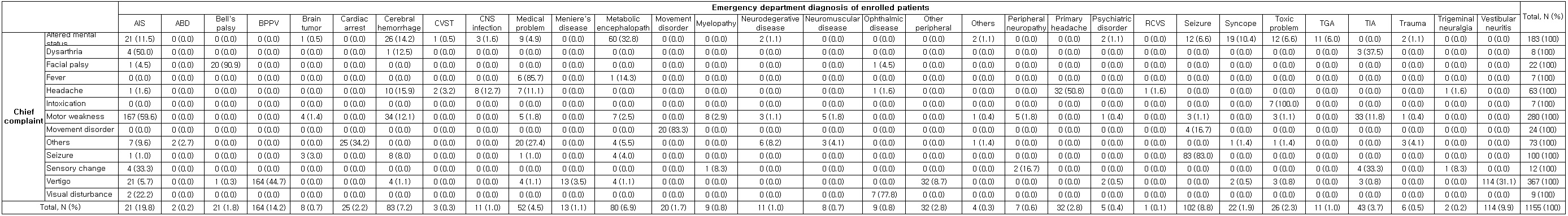

Supplement: Supplementary file 1 [file ijerph-18-09461-s001.zip › Supplementary__Table S1.jpg]
